# Notes and News

**Published:** 1888-04-28

**Authors:** 


### NOTES AND NEWS.

COLLECTED AND COMMUNICATED.

A hospital for 100 beds is proposed to be built at Paisley on the Abbey Poorhouse grounds at Craw Road.

Dr. Scanes Spicer has been appointed Physician to the Department for Diseases of the Throat in St. Mary's Hospital, Paddington.

The Marchioness of Londonderry visited the City of Dublin Hospital on the 13th inst., and made the following entry in the visitors' book: "It has given me much pleasure visiting this hospital and seeing how comfortable the patients seemed in every way, and also the clean and excellent appearance of the nurses."

Dr. J. F. Shekleton, secretary to the Bristol Royal Infirmary, has sent to the *Bristol Mercury* the following letter which he received from a patient who was treated in the infirmary twenty-two years ago, and has since been in the Royal Navy till recently: "Bristol, April 10th. Sirs,—I hope I am not doing nothing to offend you when I explain that my reason for writing to you is this: I see in the *Daily Press* that instead of people help supporting the noble institution, that the subscriptions of late has fallen off. I beg of you to let me have a box of yours in my house, and although my house is not one of the big ones in the city, yet I can promise that I can get a little towards so noble a charitable institution; and I can't afford to give a lot myself, yet I think that I can do a little towards it, and if every publican in Bristol was to see it in the same light, it would be much better for one of the best of our institutions in Bristol, and I remain, dear Sir, yours, &c." Well done, Old Salt!

At a public meeting held in the Temperance Hall in connexion with the charges made against the management of the Leicester Provident Dispensary, the following resolution was moved and carried unanimously: "That this meeting views with surprise and alarm the action of the subscribers at the annual meeting of the Leicester Provident Dispensary in refusing to institute a strict and impartial investigation into the charges made against the management of the institution; that inasmuch as this would stimulate the belief in the reality of these charges, and ultimately lead to the ruin of the institution, this meeting requests his Worship the Mayor—who presided at the annual meeting, and who gave the assurance that if these charges were properly formulated such an inquiry would become imperative—to take immediate steps to this end; that this meeting would also express the conviction that no such investigation would be satisfactory at which the members and the medical profession are not adequately represented; and that a copy of this resolution be sent to his Worship the Mayor."

Arrangements are already being made in Birmingham for the street collection by ladies on Hospital Saturday, May 12th. Last year the Hospital Saturday Fund realized £6,675, a considerable proportion of which was brought in by the ladies' collections.

The Mayor of Grimsby (Alderman Veal) offered some time ago to give £500 to build a new wing to the Grimsby Hospital. The plans for the new wing are now ready, and have been approved of; but if carried out, they will cost several hundreds of pounds more than the sum already promised. In this dilemma the Mayor has generously come forward and promised to defray the additional cost.

Mr. A. W. Webster, of Sunderland, who contributed several valuable articles to our columns dealing with the methods of raising income for hospitals available for working men, will read a paper at the Memorial Hall on the 30th instant. The subject of the paper, of which we publish a synopsis elsewhere, is, "How to extend the usefulness of the Hospital Saturday Movement, with special reference to Workmen's Subscriptions." Mr. Webster, from his large experience, is well entitled to speak on such a topic, and it is very desirable that workmen should be present in large numbers. It may be hoped also that managers and secretaries will find time to attend and to take part in the discussion. The time has now fully arrived for a general effort to make hospitals and their work extensively known and universally popular.

The Countess of Strafford and the other ladies of the Committee of the Queen's Jubilee Offering are bringing their arduous and protracted labours of loyalty and love to a successful close. The offering reached the substantial sum of £84,116, and was contributed to by more than three millions of her Majesty's subjects. Of this large sum £70,000 will be devoted to the training of nurses for the sick, and £10,000 to the completion and erection of the statue of the late Prince Consort, which is an enlarged replica of the celebrated statue by Baron Marochetti, at Glasgow, and is to be erected, when completed, in Windsor Great Park. A summary of the numbers of the contributors and the amounts shows that in England 2,206,122 persons gave £62,870; in Scotland, 415,165 gave £10,371; in Ireland 158,620 gave £3,685, and in Wales 172,946 gave £3,118. Contributions were also received from Jersey, Guernsey, Alderney, Sark, the Isle of Man, the Young Women's Christian Association, and her Majesty's foreign dependencies. £4,116 will be devoted to the purchase of a jewel for the personal adornment of her Majesty. This amount is not, as may be imagined, deducted from the subscriptions, but is the interest on the £80,000, which was invested immediately the various sums which were collected were forwarded to the head office in London. In addition to this the whole of the working expenses of the fund were also paid in the same manner—*i.e.*, by the interest on the capital, so that beyond the money expended on the statue, the whole of the great surplus £70,000 will be spent entirely on the training of nurses. It is worthy of note that of the four divisions of the United Kingdom, Scotland, both in the number of her contributors and in the total amount contributed relatively to her population, stands at the head. We are credibly informed that in many instances the most touching manifestations of devoted loyalty towards her Majesty were displayed in remote and outlying districts, amongst the poorest of the Scottish peasantry. Numbers who were absolutely without money tramped for miles across moor and bog with eggs, butter, and other such things as could be readily disposed of for a trifle, in order to add their mite to the Jubilee Offering. The Countess of Strafford, and all who have had charge of the fund, are to be sincerely congratulated on so happy and successful a termination to their labours.

The following vacancies are announced this week, the amount of salary in each case being given after the name of the institution :—

**Resident Medical Officers.**

Birmingham General Dispensary. Resident Surgeon.—£150 per annum, and £30 extra for cab hire.
Birmingham General Hospital. Assistant House Surgeon.—Board, etc.
Birmingham General Hospital. Resident Surgical Officer.—£130 per annum, and board.
Hertford British Hospital, Paris. House Surgeon.
Hospital for the Paralysed and Epileptic, Queen Square, W.C. House Physician.—£50 per annum, with board.
Liverpool Infirmary for Children. Assistant House Surgeon.—Board.
Newport and County Infirmary. House Surgeon.—£100 per annum, with board.
Royal South Hants Infirmary, Southampton. Assistant House Surgeon.—Board.
Warwick County Lunatic Asylum, Hatton, near Warwick. Assistant Medical Officer.—£100 per annum, with board.

**Non-resident Medical Officers.**

Borough of Brighton. Medical Officer of Health.—£500 per annum.
Bristol Royal Infirmary. Honorary Assistant Physician.
Durham County Hospital. Honorary Surgeon.
Durham County Hospital. Honorary Surgeon-Dentist.
Liddell Provident Dispensary, Jarrow-on-Tyne. Medical Officer.—£200 per annum.
London Skin Hospital, 47, Cranbourne Street, W. Assistant Medical Officer.
London Throat Hospital, Great Portland Street, W. Surgeon.
Seamen's Hospital Society. Visiting Physician.

Lord Robartes presided at the festival dinner of the North London Hospital for Consumption on Wednesday at the Hotel Metropole. The annual report of the institution, which has just been published, shows among other things that the income has nearly doubled within the last four years. In 1884 it stood at a little over £3,000; in 1887 it reached a total of nearly £6,000. This speaks volumes for the energy of the officials, as well as for the increasing popularity of the hospital. Mount Vernon is one of the finest sites in London for a consumption hospital. It is high and dry, close to Hampstead Heath and the open country beyond. The hospital is designed for 120 beds. At present, for want of means, it only has 34. There is a considerable mortgage on the building and site, so that it is not desirable to extend immediately. But in view of the admirable situation of the hospital and the urgent necessity for increased accommodation for consumptives in and around London, it is a very great pity that extension cannot be proceeded with. There are all the resources of treatment at command, and a little added means would make them available for three times as many patients as are now received. If some generous man of wealth could see his way to a donation of £50,000, the hospital might at once become one of the largest and best special hospitals in the kingdom.

There can be little doubt that it is desirable in the interests of all the hospitals that the responsible officers placed in charge of each and all the departments should receive training for their work before undertaking the details of it. The governors of the Middlesex Hospital, one of the best managed in London, have recognised this fact by selecting Mr. F. Clare Melhado to succeed Mr. Bartholeyns in the office of "secretary superintendent." Mr. Melhado, like many of the ablest hospital secretaries, commenced with a business training, followed by an active experience as honorary secretary of various charities in connexion with All Saints' parish, Paddington. Mr. Melhado has devoted himself entirely to the Middlesex Hospital for the last nine years, and the success of the Residential College, and also of the "Trained Nurses' Institution" (of both of which he was secretary from the commencement), is largely due to his zeal and energy.

We have already said that the Middlesex is one of the best-managed hospitals in London, and a visit to the new out-patients' department would go far to convince a stranger that this statement was only just. Here he would find the refreshment bar for out-patients, which has been satisfactorily worked for two years under the management of Mr. Melhado. The London Hospital has just taken steps to supply the out-patients with light refreshments during the hours of waiting; an arrangement which ought to find a place in the out-patient department of every large hospital. Our Commissioner has already described in these columns the weariness of the out-patients' room to a healthy person, who remains in it from the time it is opened until the departure of the last patient. To be ill under such circumstances must add greatly to the burden, and no hospital board would probably hesitate for a moment to institute a refreshment bar if all, or at any rate a majority, of its members could be put upon a course of out-patient waiting such as all out-patients have to bear.

St. Andrew's Convalescent Home, Folkestone, is an admirable institution of its kind. During last year 1,428 patients were received there—496 men, 706 women, and 227 children. About half the patients come from London, the remainder from all parts of the county. The average stay of each patient was twenty-three days, and the cost of maintenance 13*s.* per week. The home is situated on East Cliff, and everything in the management is so pleasant and homely that the feeling of being "in hospital" is wholly taken away.

Miss Margaret Ewing, the Lady Superintendent, sends us the following account of the Convalescent and Home of Rest for Women in Business and others: "This useful home (Heytesbury, Wilts) is again open for the summer season. Last year between forty and fifty patients, from various parts of the country, found a pleasant recruiting place here, and nearly all were benefited by the change. The neighbourhood is pretty, walks varied over pleasant downs, and the air most invigorating. The home is large and roomy, containing twelve beds, and the happiness and comfort of the inmates is especially studied. Admission is 8*s.* weekly, including doctor and medicine. Subscribers of one guinea can send a patient to the home for a month. Further particulars and rules upon application to the Lady Superintendent.

Miss Lucy R. Poole, Lady Superintendent of The Firs Home, Bournemouth, has sent us the report of that institution, which shows it to be of an altogether exceptional character. It is intended for the reception of cases of advanced consumption, for those cases, in fact, which are practically incurable, and which most hospitals for consumption refuse to accept. The climate of Bournemouth is known to be particularly favourable to consumptive patients, but the cost of living there is prohibitive to many sufferers. At the Firs Home, however, patients are received and carefully tended on payment of 10*s.* 6*d.* a-week, a sum which it is obvious cannot cover all necessary expenses. Therefore, for at least two-thirds of its income the Home is dependent on voluntary contributions. During last year there were 54 patients treated at the Firs Home, of whom 23 died, and 11 were discharged as being so far recovered that they were no longer eligible for residence in it, while at the end of the year 20 remained in the house. Considering the expense attendant on the treatment of consumption, the Home must be looked upon as managed economically, when it is stated that the total expenditure amounted to £1,261 12*s.* 1*d.*; but this unfortunately exceeded the receipts by £90, the demand on the resources of the institution having been great. This fact shows that the Firs Home meets a felt want, and while it emphasises its claim to larger support from the charitable public, it also suggests how advisable it would be to establish similar homes in other health resorts for sufferers from a disease that may be looked upon as a national scourge.

